# A Review of the Actions of Endogenous and Exogenous Vasoactive Substances during the Estrous Cycle and Pregnancy in Rats

**DOI:** 10.3390/ani9060288

**Published:** 2019-05-29

**Authors:** Luisauris Jaimes, Raúl Vinet, Marcela Knox, Bernardo Morales, Julio Benites, Claudio Laurido, José L. Martínez

**Affiliations:** 1 Faculty of Chemistry and Biology, University de Santiago de Chile, Estación Central 9160020, Chile; luisauris.jaimes@usach.cl (L.J.); bernardo.morales@usach.cl (B.M.); 2 CMBi, Faculty of Pharmacy, Universidad de Valparaíso, Valparaíso 2360102, Chile; raul.vinet@uv.cl (R.V.); marcela.knox@gmail.com (M.K.); 3 Regional Centre for Studies in Food and Health (CREAS, Grant R17A10001), Valparaíso 2362696, Chile; 4 Faculty of Health Science, Universidad Arturo Prat, Iquique 1100000, Chile; julio.benites@unap.cl; 5 Vice Chancellor of Investigation, Development and Innovation, Universidad de Santiago de Chile, Estación Central 9160020, Chile

**Keywords:** vascular reactivity, estrous cycle, vasoactive substances, cardiovascular diseases, endothelium, endothelial cells, pregnancy

## Abstract

**Simple Summary:**

The vascular endothelium plays an essential role in regulating cardiovascular homeostasis by controlling the vascular tone. However, it is known that variations of sex hormones in the female during the reproductive cycle have a significant impact on the homeostasis of the female cardiovascular system. Therefore, this review shows an overview of how the vascular smooth muscle response is altered in the presence of some endogenous and exogenous substances with vasoactive actions during the estrous cycle and during pregnancy in rats. We performed a systematic review of the literature using the Web of Science, PubMed, and Scielo database through multiple combinations of terms. We selected only those terms that coincided with the objectives of this study. The result of the review shows that several studies have observed both vasoconstrictive and vasodilatory responses induced by vasoactive substances during the estrous cycle and in ovariectomized and pregnant rats. The understanding of these effects is essential to optimize and develop new treatments for some vascular pathologies.

**Abstract:**

Vascular endothelium plays a key role in regulating cardiovascular homeostasis by controlling the vascular tone. Variations in sex hormones during the reproductive cycle of females affect the homeostasis of the cardiovascular system. Also, the evidence shows that estrogens show a cardioprotective effect. On this basis, this study describes some vascular responses induced by vasoactive substances during the estrous cycle in rats. We obtained the information available on this topic from the online databases that included scientific articles published in the Web of Science, PubMed, and Scielo. Many investigations have evaluated the vasoactive response of substances such as acetylcholine and norepinephrine during the estrous cycle. In this review, we specifically described the vascular response to vasoactive substances in rats during the estrous cycle, pregnancy, and in ovariectomized rats. In addition, we discussed the existence of different signaling pathways that modulate vascular function. The knowledge of these effects is relevant for the optimization and development of new treatments for some vascular pathologies.

## 1. Introduction

The vascular endothelium is a tissue of mesodermal origin made up by a monolayer of polygonal cells of 10–50 μm thickness called endothelial cells (ECs). ECs are oriented longitudinally toward blood flow, covering the interior of all vessels [1]. The endothelium is a metabolically active tissue with great functional versatility playing a key role in the regulation, maintenance, and control of the vascular tone, through the production and release of various substances [2,3,4]. The regulation of the vascular tone and reactivity involves several factors, so that the balance resulting from the action of endothelial factors that induce vasoconstriction and cell proliferation (e.g., Ang-II, endothelin (ET), thromboxane A2 (TXA2), superoxide anion) and the factors inducing vasodilation and antiproliferation (e.g., nitric oxide (NO), prostacyclin (PGI2) and hyperpolarizing factor (EDHF)) is essential for the maintenance of the vascular tone [5,6,7]. Also, the vascular endothelium is responsible for the modulation of vascular cell growth, regulation of leukocyte and platelet adhesion, selective capillary transport, regulation of plasma lipids, and maintenance of a non-thrombogenic capillary surface [2,3,8,9,10,11].

ECs, in their normal state, act by regulating the balance between procoagulant and anticoagulant factors, for example, elaborating thrombomodulin and inhibiting the formation of the tissue factor (TF) that avoid the formation of thrombus. They also inhibit inflammation by releasing PGI2, preventing the adhesion of leukocytes, and thus maintaining vascular homeostasis [12,13,14]. The imbalance in the bioavailability of some of these modulators or loss in the integrity of the vascular endothelium can lead to endothelial dysfunction associated with inflammation, vasoconstriction and increased vascular permeability and the appearance of associated cardiovascular pathologies [15,16,17].

Sex hormones are specific in men and women and influence many functions. The relationship between sex hormone levels and cardiovascular diseases has been demonstrated [18]. The incidence of these pathologies is lower in premenopausal women than in men of the same age, later equaling during menopause [19]. In this sense, the role of estrogens in the vascular system has received considerable interest, since there is experimental and clinical evidence that they have beneficial or protective effects on the cardiovascular system in females [20,21,22,23,24,25,26]. Evidence confirms that the hormonal status of women fluctuates with each reproductive cycle and during pregnancy [27,28,29]. However, little information is available on the mechanisms of control of reactivity during the ovarian cycle and pregnancy, especially regarding how reproductive hormones regulate their functions. This information is an important milestone for a thorough understanding of the homeostasis of blood pressure.

The discovery of the endothelial function in the acetylcholine (ACh)-induced vasodilation represented a milestone in the biological sciences and also had an important consequence in preparing the isolated rat aorta as an experimental model [30]. Since then, the evaluation of changes in the vascular reactivity in the isolated rat aorta model has been a widely used method [30]. Also, the estrous cycle of the rodent has turned out to be an ideal model for cognitive and reproductive research since most of the cytological data are well characterized [31,32].

Because of these advances, some aspects of the vascular function have been studied in depth, while other issues remain unexplored. An important issue to consider is the decrease of sex hormones in aging, which is often associated with an increased risk of cardiovascular disease, the leading cause of death worldwide. The knowledge of how variations in sex hormones are involved in the development of vascular disorders is essential to find better strategies to prevent and treat cardiovascular diseases [33].

Various endogenous and exogenous substances affect the vascular reactivity in different ways; most times, the specific effect of such agents during the ovarian cycle or human pregnancy is unknown [31,32]. Therefore, this review aims to show a general overview of how the vascular response is altered in the presence of endogenous and exogenous vasoactive substances under the action of the estrous cycle and pregnancy using rats as an experimental model.

## 2. Materials and Methods 

We performed a systematic review of the scientific literature using the Web of Science, PubMed, and Scielo databases through multiple combinations of MeSH terms: “Vascular Reactivity”, “Endothelial Factors”, “Rat Estral Cycle”, “Pregnancy”, “Vasoactive Substances” and “Vasoactive Natural Extracts”. We limited the search to studies in rats. We excluded articles written in a language other than English or Spanish. We obtained over 6000 articles that were analyzed and subsequently those that correspond to the objectives of this study were selected. Following this criterion, we chose and used 182 articles as a reference for this review.

### 2.1. Endothelial Factors Involved in the Modulation of Vascular Tone

Currently, it is known that the maintenance of the vascular tone and its regulation is due to the production and release of various endothelium-dependent factors. These factors, whether released by autocrine or paracrine mechanisms, are the determinants of the active participation of the endothelium in the vascular tone. According to their function, they are grouped into endothelial factors that induce vasoconstriction and proliferation, and factors that produce vasodilation and antiproliferation (see Table 1) and are described below:

#### 2.1.1. Angiotensin II (Ang-II)

Ang-II is a vasoactive peptide hormone of relevance for maintaining the structural and functional integrity of the arterial wall, and for its role in regulating the renal, vascular, and cardiac function. This vasoactive peptide comes from its precursor angiotensin I (Ang-I) which is transformed into Ang II through the action of the dipeptidyl carboxypeptidase enzyme located in the membrane of ECs, also called angiotensin conversion enzyme (ECA). The effects of Ang II are related to the modulation of synaptic transmission, stimulation of vasopressin secretion, vasoconstriction, and stimulation of aldosterone secretion by the adrenal cortex. Besides, it can act in an endocrine manner to maintain blood pressure and electrolyte balance; it can also regulate highly specific cellular functions, such as cytokine secretion and cell proliferation [34,35,36]. These actions are mediated by complex signaling systems activated by the binding of the hormone to specific receptors, namely AT1 and AT2 [37,38,39,40]. In the vasculature, the AT1 and AT2 receptors are present in large numbers, concentrating mainly on smooth muscle cells; these receptors are in a low number in the adventitia, and practically they are not expressed in ECs [34]. AT1 and AT2 differ in the G protein they preferentially activate and in the variety of signals they initiate. Ang II is involved in various cardiovascular diseases, maintaining arterial hypertension by vasoconstrictor mechanisms, stimulating the sympathetic nervous system, increasing the release of aldosterone and increasing the production of superoxide anions, mainly in the endothelium and the adventitia through NADPH oxidase-linked membrane [41,42,43]. Recently, it was demonstrated that the ECs of cava and portal veins show important properties to be activated by Ang II, probably influencing the whole circulatory system [44].

#### 2.1.2. Endothelin (ET)

Endothelins are a family of vasoconstrictor peptides that contribute to regulating the vascular tone and cell proliferation constituted by three isoforms of 21 amino acids with four cysteine residues, establishing two disulfide intramolecular bridges, forming an unconventional semi-conical structure [45,46]: Endothelin-1 (ET-1), endothelin-2 (ET-2) and endothelin-3 (ET-3) differing in their potency of vasoconstrictive action [47]. The biological effects of ET-1 are relevant in the maintenance of the basal vasomotor tone and blood pressure in humans [48,49,50,51,52]. They have direct mitogenic effects on smooth muscle cells, stimulating the production of cytokines and growth factors [26,53,54]. They also induce the formation of extracellular matrix proteins and fibronectin and potentiate the effect of transforming the growth factor-beta (TGF-b) and platelet-derived growth factor (PDGF) [26,53]. On the other hand, vasodilators such as NO and PGI2 act by inhibiting the production of ET-1 through common mechanisms involving the production of cyclic guanosine monophosphate (cGMP). The same function fulfills the auricular natriuretic hormone, inhibiting the basal ET production. These same hormones inhibit the mitogenic and vasoconstrictor effect stimulated by ET [55,56]. It has been proven that the synthesis of ET-1 is stimulated and associated with various cardiovascular risk factors, the main ones are high cholesterol levels, low-density lipoproteins, and glucose, estrogen deficiency, obesity, pro-coagulant mediators similar to thrombin. Also, vasoconstrictors, growth factors, cytokines, and adhesion molecules also stimulate endothelin production [52,57,58,59,60,61]. 

#### 2.1.3. Thromboxane A2 (TXA2)

TXA2 is an eicosanoid resulting from the transformation of the arachidonic acid (AA) derivative by the enzyme TXA2 synthase. It has been shown that TXA2 is a potent platelet aggregator and vasoconstrictor produced by the vascular wall and its biological actions involve platelet aggregation, contraction of muscle cells [62], regulation of cell migration processes endothelial, angiogenesis and tumor metastasis [26,63]. Such functions explain their participation in the process of hemostasis, while in the respiratory system it is a powerful bronchoconstrictor. The specific receptor for TXA2 is called TP and has two isoforms called α and β, through which TXA2 exerts its actions. They are distinguished by their carboxy-terminal end located inside the cell. This fact explains the multiple transduction pathways of signals activated by this receptor through different G proteins [64,65,66,67]. The release of TXA2 and other PGs vasoconstrictors can be stimulated by different vasoconstrictive agents such as ET, 5-HT, NA, and A-II, as well as by vasodilator agents such as ACh or by mechanical actions on endothelial cells [68,69,70]. It has been described that both endothelial cells [61,69] and the smooth muscle [71,72] synthesize TXA2. The synthesis of TXA2 is increased in a variety of cardiovascular disorders, in which TXA2 is implicated as a key cellular mediator [73]. TXA2 participates in the endothelial dysfunction associated with different cardiovascular risk factors such as diabetes and hypertension [74,75]. The inhibition of the synthesis of TXA2 by aspirin has been associated with a decrease in mortality caused by cardiovascular diseases [73]. Recently, it has been proposed that the interaction between LPA/LPA1 and TXA2/TP pathway plays a significant role in vasoregulation, hemostasis, thrombosis, and vascular remodeling (LAP = Lysophosphatidic acid; TP = Thromboxane prostanoid) [76].

#### 2.1.4. Reactive Oxygen Species (ROS)

The generation of reactive oxygen species (ROS), such as superoxide anion (O_2_-), is generally produced in all tissues [77,78]. However, the presence of ROS in the vasculature is a consequence of the abnormal generation and/or an inability of the endogenous reducer and antioxidant systems to eliminate these reactive species [79]. These species can exert their action directly on the vascular system or serve as a substrate for the formation of other ROS, such as hydrogen peroxide (H_2_O_2_), peroxynitrite (OONO-), hypochlorous acid (HOCl) and the hydroxyl radical (OH.), among others.

In vascular cells, potential sources of O_2_- include the mitochondrial electron transport chain and the participation of nicotinamide adenine dinucleotide/nicotinamide adenine dinucleotide phosphate (NADH/NADPH) oxidase, xanthine oxidase and NO endothelial synthase (eNOS) [80,81,82,83]. However, the NADH/NADPH oxidase system is the primary source of O_2_-vascular and comprises membrane-bound oxidases that use NADH and NADPH as substrates [84,85,86]. In non-pathological conditions, the generation of ROS by eNOS or mitochondria is limited, and in the case of mitochondria, it is probably more related to signaling pathways [87,88]. However, there is significant evidence pointing to the generation of ROS by decoupling eNOS; this situation arises when NADPH consumption is not correlated with a stoichiometric production of NO, what is called decoupling of the eNOS [89,90,91]. This phenomenon is caused by several factors that can alter the transfer of electrons in NOS, so that the amount of NADPH required to generate NO is relatively higher, primarily explained by the reduction of other substrates mainly molecular oxygen to produce superoxide [79].

These species have an unpaired electron, so they are highly reactive. Like these species, NO has an unpaired electron so that it can react with them through a chemically spontaneous and irreversible reaction [92,93]. When O_2_- reacts with NO, it inactivates, hindering vasorelaxation and inducing apoptosis in endothelial cells, favoring the appearance of thrombotic phenomena and promoting the adhesion of different cells to the endothelium. Additionally, the product of the reaction, OONO-, is a powerful oxidant that produces critical biological effects such as a protein nitrosylation [26,93,94].

The participation of ROS in the vasorelaxation has been demonstrated in at least two ways: First, in the interaction with NO and second, through the direct effects of H_2_O_2_. It is well established that the NO derived from the endothelium undergoes a rapid reaction with O_2_- reducing its bioavailability [95]. A growing number of experimental evidences in animals and humans suggest that the oxidative inactivation of NO plays an essential role in several vascular pathologies such as hypertension, diabetes, hypercholesterolemia, and arteriosclerosis [96,97,98,99].

#### 2.1.5. Nitric Oxide (NO)

NO is a free radical with an unpaired electron that can participate in several reactions, acting as a weak oxidant or reducer [100,101]. Its neutral charge allows it to diffuse freely through the biological membranes, explaining its potential to act in different biological targets [102,103]. NO can react with O_2_- forming peroxynitrite (ONOO-), a potent oxidant involved in the oxidation of proteins under physiological conditions [94].

The synthesis of NO is carried out by a family of enzymes called nitric oxide synthases (NOS), of which three isoforms exist, exhibiting different characteristics reflecting their specific functions in vivo [100,104,105,106]. There are two constitutive isoforms called eNOS or endothelial, present mainly in endothelial cells [107] and nNOS or neuronal present mainly in neurons. Both isoforms are dependent on Ca^2+^ and calmodulin [108]. The third isoform, called iNOS, is inducible and is independent of Ca^2+^ and calmodulin and is present in different cell types. iNOS is expressed in abnormal cellular processes such as heart failure [109]. Also, iNOS is induced by cytokines and inflammatory agents determining high NO levels [26,110,111,112]. Furchgott et al. (1980) [113], elegantly demonstrated that the endothelium was instrumental in the vascular smooth muscle relaxation. In rats, Rees et al. (1989) [114], found that blocking the formation of NO with the inhibitor L-NMMA resulted in an immediate rise in blood pressure and that the subsequent administration of the L-arginine substrate reversed this effect. NO also plays a role in the endothelium-dependent venous relaxation [115]. 

#### 2.1.6. Prostaglandins

Prostaglandins, like thromboxanes, are cellular mediator eicosanoids synthesized from AA by the activation of cyclooxygenases (COX) [116,117]. The (COX) are bifunctional enzymes that have two catalytic activities and a cyclooxygenase which incorporates two molecules of O_2_ to AA forming PGG2, and another peroxidase, which catalyzes the reduction of PGG2 to PGH2 [118] generating prostanoids, lipid mediators of inflammation with autocrine and paracrine action, including both prostaglandins (PGs) and thromboxanes (TXs). There are two isoforms of the COX enzymes: COX1, a constitutive enzyme present in most of the body’s cells and that regulates the basal homeostasis of prostanoid synthesis [119,120], and COX-2, an inducible enzyme against inflammatory stimuli such as the action of pro-inflammatory cytokines, bacterial lipopolysaccharides (LPS), growth factors and tumor-promoting agents [117,121,122].

Prostaglandin I2 (PGI2) is one of the primary metabolites of the COX pathway. It was first discovered by Needleman and Vane in 1976 [123,124]. PGI2 is produced in ECs and the lung parenchyma by the action of the enzyme prostacyclin synthase on prostaglandin H2 [116,125,126,127]. The main action of PGI2 is to avoid the aggregation of platelets; however, it is also an effective vasodilator [128]. PGI2 has the opposite effect of TXA2, suggesting a homeostatic mechanism between these two hormones. It has been shown that the release of PGI2 depends mainly on the exit of Ca^2+^ from intracellular deposits [129]: At low concentrations, PGI2 activates its specific IP receptor producing vasodilation by reducing the intracellular concentration of Ca^2+^ in smooth muscle cells by the action of cAMP after the activation of adenylate cyclase (AC). On the contrary, at high concentrations, PGI2 can activate IP receptors and produce vasoconstriction [130,131,132]. It has been observed that the stimulation of PGI2 synthesis by factors such as Ang-II, acetylcholine (ACh) or bradykinin (Bk), and products released from platelets such as 5-HT, interleukin-1 and platelet-derived growth factor (PDGF) [133], NO [134], ROS [135] and the prostanoids themselves [136,137], indicates a rich interaction between different cellular mediators [26,138]. Additionally, the cardioprotective effect of PGI2 has been related to a protective action against atherogenesis and heart disease, because PGI2 decreases cholesterol synthesis and reduces the activity of LDL receptors in leukocytes [139]. Likewise, it has been described that some of its analogs inhibit the progression of atherosclerosis in the aorta of hamsters and rabbits [26,140,141].

On the other hand, other eicosanoids that give rise to AA are leukotrienes (LTs) and lipoxins (LXs) through a pathway mediated by lipoxygenases (LOX); besides, hydroxyeicosatetraenoic acids (HETEs) and epoxyeicosatrienoic acids (EETs) are generated by a cytochrome P 450-mediated pathway (CYP450). Concerning the route mediated by LOXs, the three main enzymes involved are the 5-, 12- and 15-lipoxygenases (5-LOX, 12-LOX, 15-LOX) present in leukocytes, platelets, and endothelial cells, respectively [142]. LTs act as lipid mediators with the paracrine action involved in inflammatory and immune processes mediated by specific rhodopsin-receptors coupled to G-protein [117,143].

The cytochrome P450 family (CYP450) are enzymes anchored to the cell membrane with a heme group in their structure. These enzymes catalyze the conversion of fatty acids, including AA, to various derivatives, including EETs obtained from AA by the action of CYP2C and CYP2J enzymes or hydroxylated derivatives such as HETEs mediate by the CYP4A enzyme [142]. It has been suggested that the EETs could act as EDHF with the vasodilator action. One example is the conversion of AA to EETs in the vascular endothelium, with anti-inflammatory activity and anti-platelet aggregation effect, while, in the vascular smooth muscle it is hydroxylated and converted to 20-HETE with vasoconstrictive effect. The action of both compounds contributes to maintaining vascular homeostasis [143].

#### 2.1.7. Endothelium-dependent Hyperpolarizing Factor (EDHF)

The EDHF group includes epoxyeicosatrienoic acids (EETs), which respond to stimuli such as ACh, Bk, and shear stress, causing the opening of K^+^ channels [144,145]. EETs correspond to metabolites of AA in which the cytochrome P-450 is involved [146]. They are produced by the endothelium and contribute to the regulation of the vascular tone by inducing relaxation through a hyperpolarization of the smooth muscle membrane [147]. Hyperpolarization and relaxation produced by EDHF seem to be due to an increase in the conductance of the K^+^ in the vascular smooth muscle membrane, through the Ca^2+^ or ATP-dependent K^+^ channels [148], depending on the vascular bed [26]. Several studies suggest that EDHF is particularly important in response to endothelium-dependent vasodilator agents in coronary and mesenteric circulation [149]. Their participation is modified in pathophysiological situations in which there is a decrease in other endothelial factors such as NO [150]. Endothelial cells induce smooth muscle hyperpolarization in a contact-dependent manner with H_2_O_2_ [151], K ions [152], CNP [153], H_2_S [154]. Putative diffusible candidates for the EDHF activity have increased over the past 15 years [155]. Besides, EDHF appears to be the predominant endothelial factor in the vascular resistance in women, a response influenced by sex [19].

Physiologically, a lower bioavailability of NO, alterations in the production of prostanoids (including PGI2, TXA2 and/or isoprostanes), deterioration of endothelium-dependent hyperpolarization, and a higher release of ET-1, can contribute individually or associated with endothelial dysfunction. Endothelial dysfunction can be understood as an endothelium situation where ECs do not adequately fulfill the functions mentioned above, either in basal or stimulated conditions.

According to the above, and based on the evidence, it can be indicated that the ECs produce various bioactive compounds, including products generated by the enzymes eNOS and COX, capable of regulating the vascular tone. It is interesting to note that, through the activation of COX, vasoconstrictor prostanoids and vasodilators are produced, and both can be modulated in their synthesis by NO. This phenomenon and the diversity of interrelationships that occur between the signals that determine the vascular tone shows the importance of considering the influence of sex hormones on the cardiovascular system during the estrous cycle and pregnancy.

## 3. Estrous Cycle and Vascular Reactivity

An estrous cycle is a periodic event, with regular but restricted phases of sexual receptivity associated with the release of fit ovules to be fertilized [156]. This period typically covers between 17 and 24 days, considering 21 days as the average time, depending on the species [157,158]. In the rat, the estrous cycle has a relatively short duration of four days approximately and is divided into four phases, perfectly distinguishable by vaginal smear [159,160]: (1) Proestrous, follicular phase or regression of the corpus luteum with a duration of 12 h; (2) estrous, a period of sexual receptivity, at the end of which ovulation occurs with a duration of 12 h; (3) metestrous, where the initial development of the corpus luteum occurs with a duration of 21 h; and (4) diestrous, beginning of the activity of the mature corpus luteum with a duration of approximately 65 h [156,161]. Its short duration allows the rat to be an ideal species for the investigation of the changes that occur during the reproductive cycle [162].

**Figure 1:**
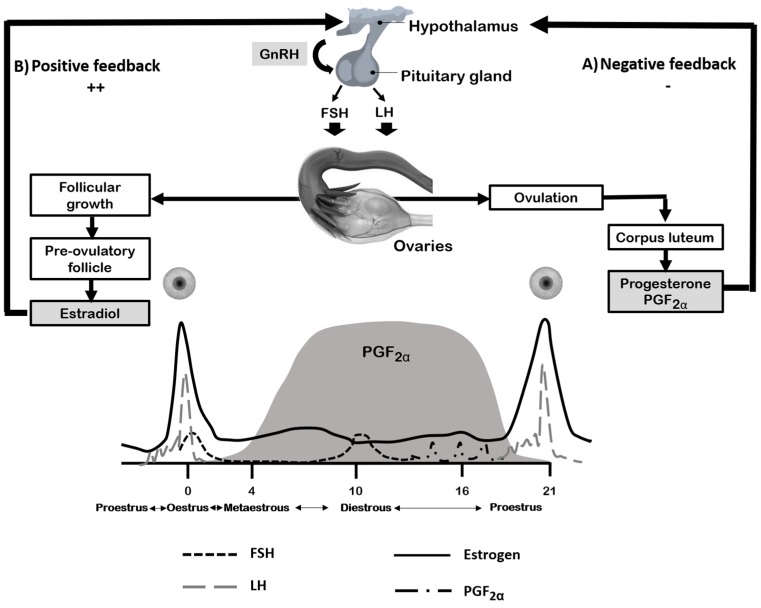
Simplified scheme of the hormonal interactions of the hypothalamus-pituitary-ovarian axis during the estrous cycle. (**A**) Negative feedback: Decreased LH concentration characteristic of the late follicular phase. The initial effects of ovarian steroids eliminate the responsiveness of the pituitary to the action of GnRH, since the secretion of these by the hypothalamus is continuous at this stage; (**B**) positive feedback: Continuation of the previous period, here is a significant increase (about 90%) in the concentration of LH. The effect produced result from the combined action of high levels of estrogen and progesterone (of ovarian origin) on the hypothalamus and of the latter (through hormonal release factors) on the pituitary. This phenomenon constitutes the endocrine event that characterizes the preovulatory period.

The hormonal regulation of the estrous cycle is under the neurological control of the hypothalamus-pituitary, ovary, and uterus axis (Figure 1). The activity begins with the pulsatile secretion of gonadotropins (GnRH) from the neurosecretory parvicellular neurons located in the arcuate nucleus, the periventricular nucleus and the preoptic area [163]. The hormone acts as a messenger, regulating each of the phases of the estrous cycle [164]. During estrous, metestrous and diestrous, the secretion of GnRH, luteinizing hormone (LH) and follicle-stimulating hormone (FSH), are at basal levels by the inhibitory or feedback-negative action of the ovarian hormones, mainly estradiol, but also, progesterone and inhibins [161,165]. The amplitude and frequency of the GnRH pulses and feedback from progesterone and estrogens control the processes of synthesis and secretion of LH and FSH from adenohypophyseal gonadotrophs. GnRH stimulates the growth of antral follicles and increases the production of ovarian steroids, which affects the secretion of LH/FSH for the benefit of negative feedback. A low frequency and high pulse amplitude of GnRH leads to the release of FSH, while a high frequency and low amplitude pulses stimulate the release of LH. However, in the female the frequency of the GnRH pulses varies during the estrous cycle, being higher during the pre-ovulatory surge of LH and lower during the luteal phase of the estrous cycle [166,167,168]. Progesterone is also produced in the corpus luteum by the action of LH and prepares the uterus to allow implantation of the embryo and to maintain pregnancy. Progesterone exerts a negative feedback effect on the hypothalamus [161].

On the other hand, during proestrous, estrogens exert a positive feedback at the level of secretion of GnRH, LH, and FSH. Among the positive actions are: (1) Cooperation with GnRH in the synthesis of its receptors at the pituitary level; (2) pituitary sensitization to GnRH (probably through a post-receptor mechanism), (3) increase of the prostaglandin receptor at the pituitary and hypothalamic level, (4) regulation of the GnRH synthesis, (5) cooperation in the appearance of the preovulatory secretion of hypothalamic decapeptide, and (6) decrease in the metabolism of GnRH in the pituitary gland, all them increasing effectiveness [168]. In the pituitary gland, estrogens carry out inhibitory and stimulatory actions on the secretion of gonadotropins by activating their receptors in the gonadotrope, among which are different types of localization and isoforms (intracellular or membrane, α or β) [161,169].

Lower amounts of estrogen are synthesized in the adipose tissue and liver from testosterone by aromatase [170], enzyme P-450 responsible for the aromatization of cholesterol for conversion to estrogen in the endoplasmic reticulum in the ovary and placenta where the synthesis of estrogens occurs, and in the adrenal gland and testes. This enzyme is also found in the vasculature and can synthesize estrogen locally for paracrine/autocrine effects in vessels [171]. Although human endothelial cells do not contain aromatase, the vascular smooth muscle cells of the aorta and the pulmonary arteries contain them [172].

These hormones play an essential role in the vascular reactivity [173], and their most prominent effects are mediated by direct actions on the endothelial function [174]. Several propositions try to explain the mechanism of action by which estrogenic hormones exert cardiovascular protection in women. One of the most accepted explanations is related to the effects of estrogen on lipid metabolism [175]. Estrogen decreases the levels of low-density lipoprotein and lipoprotein (A) in the blood [170] while it increases the cholesterol bound to high-density lipids [176,177,178]. These actions may explain the protective effect of estrogen in women against the development of atherosclerosis, a disease closely associated with menopause [175]. Atherosclerosis begins with the deposit of lipid material and blood cells, together with the formation of lesions that give rise to the fibrous plaque, which provides rigidity to the blood vessels affecting the hemodynamic properties of the circulatory system [177].

Conversely, in ECs, estrogens trigger the synthesis of factors derived from the endothelium, such as NO, ET-1, and prostacyclins [179,180]. Estrogen can bind to the α-estrogen receptor in the ECs membrane of the caveolae [181] activating the NOS [177,182]. These findings were confirmed in a clinical study where the alteration of the endothelium-dependent vasodilation induced by ACh was normalized after an estrogen replacement therapy [183]. The vascular reactivity in the estral cycle was studied in rat aortic rings [184]. Recently our group has reported that the indomethacin affects the norepinephrine-induced contraction in the vascular smooth muscle in different stages of the estrous cycle suggesting that cyclooxygenase produces an increase in contraction especially during proestrous [185].

## 4. Pregnancy and Vascular Reactivity

Pregnancy begins with the union of the egg and sperm in the tube; the egg is transferred to the uterus to complete the development of the fetus. After pregnancy, the corpus luteum, responsible for the secretion of estrogen and progesterone, increases its size and its function is maintained until the third month of gestation [29], secreting slightly higher amounts of hormones than those that occur after ovulation and second half of the menstrual cycle. From the fourth month of gestation, the placenta secretes progressively elevated amounts of these hormones, reaching a maximum at the end of pregnancy [27]. Steroids produced by the placenta come from steroidal precursors that enter from the maternal bloodstream or the fetus. Therefore, maternal estrogen levels throughout pregnancy reach concentrations thirty times higher than those found in the luteal phase. 

On the other hand, progesterone originates from the maternal cholesterol, and 90% of the progesterone produced in the placenta passes into the maternal circulation and the remaining 10% into the fetal circulation. Progesterone levels throughout pregnancy increase progressively, reaching concentrations ten times higher than those found during the luteal phase of the ovarian cycle [29]. In this sense, rats show similar changes during pregnancy, thus providing an adequate initial experimental model for pregnancy and vascular reactivity studies [186,187,188,189].

**Figure 2:**
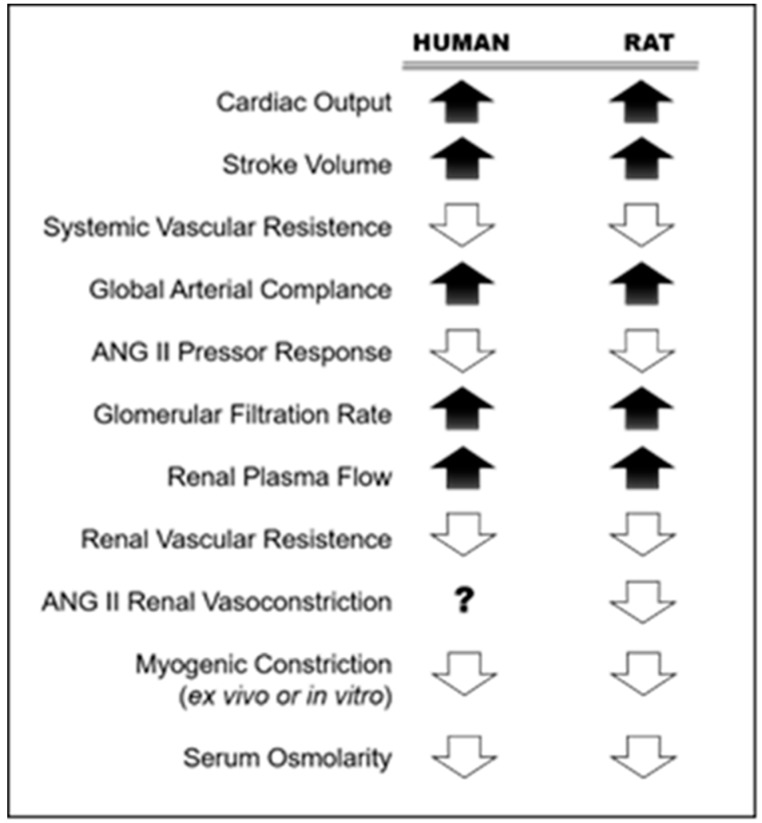
Renal and systemic vasodilation during pregnancy in human and rat. Adapted from: Conrad, 2011 [198].

During pregnancy, there are alterations in the maternal cardiovascular system that leads to the appearance of systemic vascular manifestations of the same. These changes occur rapidly to provide an efficient uteroplacental circulation that facilitates the development of the fetus [190]. It has been proposed that estrogens act as mediators of vascular changes during pregnancy due to their effects on the vasculature and its high concentration during this period. There are a number of well-described changes in the cardiovascular system (Figure 2) that include significant increases in cardiac output and blood volume with a reduction in peripheral vascular resistance [190,191,192,193,194], a significant decrease in the pressor response to vasoconstrictors administered exogenously [195], and vascular remodeling conducting to higher compliance [196]. However, the mechanisms that could explain such vascular modifications during normal pregnancy remain little known and of high relevance considering the inadequate adaptation of the cardiovascular system to pregnancy associated with hypertensive disorders during pregnancy, such as preeclampsia and fetal growth impairment [197,198].

## 5. Actions of Exogenous Vasoactive Substances on The Endothelial Function during the Estrous Cycle and Pregnancy

The difficulties associated with carrying out in vivo studies in humans have led researchers to examine the vascular changes that occur during pregnancy and the ovarian cycle through indirect methods. These have included animal models, mainly rats, and have focused on understanding the effect of vasoactive substances during the estrous cycle. Regarding pregnancy, to date, most investigations have focused on the exogenous administration of estrogens to non-pregnant animals where estrogen levels are close to those of pregnancy [199]. Likewise, the results have been contradictory with some reports of attenuation of the pressor response capacity [200,201,202,203]. However, other studies have shown no effect [204]. The differing data could be due to methodological differences including dose used, type of administration (acute versus chronic), duration of oophorectomy before administration of steroids and heterogeneity of the vascular bed [199]. In this sense, the actions of substances of non-estrogenic origin on the vascular endothelium have also been studied, and the effects of pregnancy and the influence of the estrous cycle on its effects have been determined. Table 2 summarizes some results.

## 6. Conclusions

After a thorough review of the literature, it is possible to conclude that sex hormones, mainly estrogens, play an important role as modulators of the vascular tone through the vascular endothelium. Evidence shows that these effects are associated with the cardio protection observed in young (premenopausal) women and in pregnancy.

Likewise, the vascular effects of some natural and synthetic products have been discussed as agents capable of modulating the vascular tone in rats. However, more research is still needed on this issue to better understand the effective impact of these vasoactive substances during the estrous cycle. Herbs have a potential therapeutic value in the prevention and/or complementary treatment of vascular disorders that may occur during human pregnancy.

## Figures and Tables

**Table 1:** Factors released by the vascular endothelium (Adapted from: De la Serna (2010) [14]).

**Vasoactive Factors**	
Vasodilators	-Nitric oxide (NO)
	-Endothelium-derived hyperpolarizing factor (EDHF)
	-Prostacyclin (PGI_2_)
Vasoconstrictors	-Endothelin 1 (ET-1)
	-Angiotensin II (Ang II)
	-Thromboxane A2 (TXA_2_)
**Growth modulators**	
Growth promoters	-Platelet-derived growth factor (PDGF)
	-Basic fibroblast growth factor (bFGF)
	-Somatomedin-1 (IGF-1)
	-Endothelin 1 (ET-1)
	-Angiotensin II (Ang II)
	-Heparan sulfate (HPS)
Growth inhibitors	-Transforming growth factor (TGF)
	-Nitric oxide (NO)
	-Prostacyclin (PGI_2_)
**Modulators of inflammation**	
Adhesion molecules	-Endothelial leukocyte adhesion molecule (ELAM) -Intercellular adhesion molecule (ICAM) -Vascular cell adhesion molecule (VCAM)
**Hemostatic and thrombolytic factors**	
	-Tissue plasminogen activator (t-PA) -Plasminogen activator inhibitor 1 (PAI-1) -Thrombomodulin

**Table 2:** Some vasoactive substances capable of modulating vascular tone in different endothelial tissues.

Vasoactive Substance	Model	Tissue	Actions in Endothelium	References
Norepinephrine (NE)	Control group: Cyclic rats Experimental group: Ovariectomized rats (OVX)	Aorta (vascular smooth muscle) Treated in isometric myography system	-The vascular synthesis of both PGE2 and PGF2a was significantly higher in the group of OVX rats compared to the proestrous. -The vascular response to NE was significantly higher in OVX rats, compared to normal cycling rats during proestrous.	[205]
Cirazoline (α_1_-Adrenergic agonist)	Pregnant, proestrous and diestrous rats	Mesenteric vascular bed Treated by perfusion	-The tone induced by cirazoline was lower in the proestrous and pregnant groups, but the increase in the tone of L-NA is higher in pregnant compared to proestrous and diestrous group. The participation of EDRF in this effect is suggested.	[206]
Methacholine (Muscarinic agonist, endothelium-dependent vasodilator)	Control group: Experimental group: Pregnant mice	Uterine and mesenteric arteries Treated in isometric myography system	-The relaxation induced by methacholine was higher in pregnant mice, in both uterine artery and mesenteric vessels, with a more pronounced effect on the uterine vasculature. -Modulation of relaxation is endothelium-dependent PGHS or NOS pathways is reinforced in the uterine arteries.	[207]
*Aspilia africana* (Ethanolic extract)	Cyclic rats	Uterine endothelium Treatment by using an oropharyngeal cannula and calibrated hypodermic syringe	-Dose-dependent decrease in duration of estrous cycle and histoarchitecture of the uterus.	[208]
*Buddleja globosa* (Ethanolic extract)	Cyclic rats Ovariectomized rats (OVX)	Uterine endothelium Treatment administered subcutaneously with hypodermic syringe	Anti-estrogenic effect of extract of *Buddleja globosa* at the highest dose evaluated.	[209]
